# Account of Diseases in an Eastern District of London, from November 20, to December 20, 1804

**Published:** 1805-01-01

**Authors:** 


### *Account of Diseases in an Eastern District of London, from November 20, to December 20, 1804.*

| | |
|---|---|
| **ACUTE DISEASES.** | |
| Peripneumonia Notha | 4 |
| Ephemera | 3 |
| Dysenteria | 7 |
| Rheumatismus Acutus | 5 |
| **CHRONIC DISEASES.** | |
| Tussis | 25 |
| Tussis cum Dyspnœa | 30 |
| Dyspnœa | 17 |
| Catarrhus | 5 |
| Hæmatemesis | 2 |
| Phthisis Pulmonalis | 3 |
| Hydrothorax | 2 |
| Hepatitis Chronica | 1 |
| Ascites | 3 |
| Amenorrhœa | 4 |
| Menorrhagia | 3 |
| Chlorosis | 4 |
| Diarrhœa | 14 |
| Paralysis | 2 |
| Rheumatismus Chronicus | 30 |
| **PUERPERAL DISEASES.** | |
| Menorrhagia Lochialis | 5 |
| Ephemera | 4 |
| Ischuria Vesicalis | 1 |
| **INFANTILE DISEASES.** | |
| Diarrhœa | 10 |
| Ascites | 1 |
| Ophthalmia | 2 |
| Vermes | 4 |

